# Regulation of alternative splicing and polyadenylation in neurons

**DOI:** 10.26508/lsa.202302000

**Published:** 2023-10-04

**Authors:** Seungjae Lee, Joseph I Aubee, Eric C Lai

**Affiliations:** Developmental Biology Program, Sloan Kettering Institute, New York, NY, USA

## Abstract

Cell-type–specific gene expression is a fundamental feature of multicellular organisms and is achieved by combinations of regulatory strategies. Although cell-restricted transcription is perhaps the most widely studied mechanism, co-transcriptional and post-transcriptional processes are also central to the spatiotemporal control of gene functions. One general category of expression control involves the generation of multiple transcript isoforms from an individual gene, whose balance and cell specificity are frequently tightly regulated via diverse strategies. The nervous system makes particularly extensive use of cell-specific isoforms, specializing the neural function of genes that are expressed more broadly. Here, we review regulatory strategies and RNA-binding proteins that direct neural-specific isoform processing. These include various classes of alternative splicing and alternative polyadenylation events, both of which broadly diversify the neural transcriptome. Importantly, global alterations of splicing and alternative polyadenylation are characteristic of many neural pathologies, and recent genetic studies demonstrate how misregulation of individual neural isoforms can directly cause mutant phenotypes.

## Abstract (precis)

Neurons generate numerous cell-specific isoforms of genes that are expressed more broadly. Here, we review mechanisms that generate neural-specific alternative splicing and 3′UTRs and their biological impacts on the nervous system.

## Introduction

Most protein-coding genes in higher eukaryotes are subject to alternative processing yielding multiple mRNA isoforms, thereby diversifying the functional transcriptome (Tian & Manley, 2017; Ule & Blencowe, 2019). The ability to generate complex alternative transcripts from individual genes is critical to the appropriate specification, differentiation and function of distinct cell types, and homeostatic responses to an array of perturbations (Vuong et al, 2016a; Olthof et al, 2022; Wright et al, 2022). Consequently, alternative mRNA processing is deregulated under diverse pathogenic conditions, and defects in some individual isoforms can cause disease (Bonnal et al, 2020; Rogalska et al, 2022). For these reasons, it is important to appreciate the breadth of alternative transcript isoforms across time and space, understand mechanisms by which specific alternative mRNA isoform choices are executed in the correct cell states and conditions, and elucidate biological consequences of failure to generate appropriate programs of alternative isoforms.

**Figure 1.**
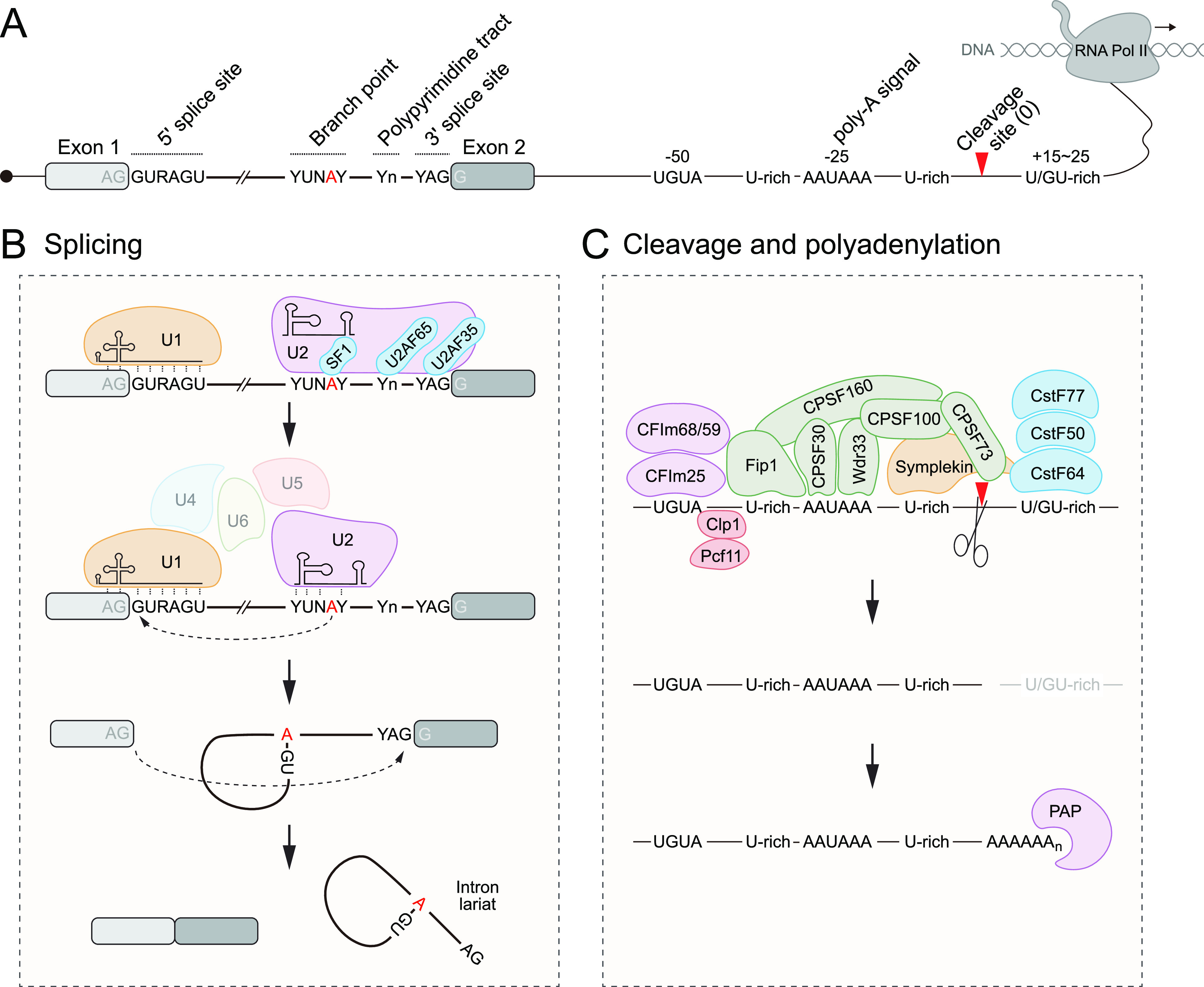
Fundamental mechanisms for mRNA processing: splicing and polyadenylation. **(A)** Primary mRNA transcripts bear a series of sequence motifs that direct splicing (left side, sequences in the vicinity of exon–intron boundaries) and cleavage and polyadenylation (right side, sequences in the vicinity of 3′ termini). **(B)** Core features of splicing. This process is defined by cis-acting sequences at the 5′ exon–intron junction (GU, often within the AG|GURAGU context), the branchpoint (YUNAY), and the 3′ intron–exon junction (AG, often within the YAG|GU context). The spliceosome contains multiple ribonucleoprotein subcomplexes that mediate different aspects of intron excision and exon ligation. Splicing is a dynamic stepwise process, and the stepwise recruitment and ejection of splicing subcomplexes is not fully detailed here for simplicity. Central players include the U1 snRNP that recognizes the 5′ intron boundary and the U2 snRNP that recognizes the 3′ intron boundary. The 5′ splice site basepairs with U1 snRNA, whereas the 3′ intron region is initially bound by SF1 (branchpoint), U2AF65 (polypyrimidine tract), and U2AF35 (3′ splice site); this transitions to basepairing of U2 snRNA around the branchpoint. The two fundamental steps of splicing are ligation of the branchpoint A to the 5′ splice site G, followed by ligation of the 3′ end of the upstream exon to the 5′ end of the downstream exon, joining the exons. This simultaneously liberates an intron lariat, which is then debranched and degraded. **(C)** Core features of 3′ end formation. The multisubunit cleavage and polyadenylation complex recognizes the presumptive 3′ end via sequence motifs, including the polyadenylation sequence (typically AAUAAA), which is often flanked by other upstream and downstream sequences. Following cleavage of the primary mRNA transcript by the CPSF73 endonuclease, the 3′ end is extended via poly-A polymerase to ensure a stable terminus, that is, protected by poly-A–binding protein.

Many classes of alternative mRNA processing include usage of different promoters, inclusion of distinct internal exons, and deployment of alternative last exons (ALEs) and/or 3′ UTRs (Fig 1A). Conceptually, these phenomena invoke choices of basal promoters, of splicing sites, and of polyadenylation sites. Different machineries are involved in identifying each of these alternative sites, and moreover, individual genes can be subject to alternative processing at multiple locations to generate combinatorial complexity. With these complexities in mind, it is notable that we lack full mechanistic understanding of several established, critical, regulators of isoform diversity. We also are far from knowing the biological importance of many such programs, which is ultimately critical to decipher their contributions to human disease.

In this review, we will focus on alternative splicing (AS) of internal and 3′ terminal exons and on alternative polyadenylation (APA) to generate distinct 3′ UTRs. We note that others have extensively reviewed general mechanisms and regulation of alternative splicing (Tian & Manley, 2017; Bonnal et al, 2020) and APA (Ule & Blencowe, 2019; Mitschka & Mayr, 2022). We direct the reader to such recent reviews for comprehensive background on these topics. Among the broad literature on these processing programs, we pay attention to exemplary cell-specific RNA-binding proteins (RBPs) that instruct isoform programs (Darnell, 2013). As for setting, we will focus on the nervous system, whose utilization of both cell-specific splicing and alternative 3′ UTR programs is particularly widespread. The diversity of neural transcriptomes is critical for the development, function, and maintenance of these unusual cells, and also exposes vulnerabilities of neurons when these mRNA-processing mechanisms go awry. Although we review classic literature and general studies on isoform generation, we will emphasize the latest mechanistic findings, technical innovations, and biological impacts of neural-specific AS and neural APA.

### Fundamentals of splicing

It was originally conceived that each gene generates an individual protein product (Beadle & Tatum, 1941). However, the molecular structure of eukaryotic genes turned out not to be that simple. First, genes can be spliced, thereby joining discontinuous segments of transcribed sequences (Berget et al, 1977; Chow et al, 1977; Klessig, 1977). Second, most genes in higher eukaryotes actually yield multiple mRNA isoforms that differ in their content of coding exons, due to differences in alternative promoter selection/5′ exons (Feng et al, 2016; Demircioglu et al, 2019) and alternative internal or 3′ terminal exons (Pan et al, 2008; Wang et al, 2008). Therefore, a single gene can generate multiple transcripts, encoding numerous protein products. Alternative 5′ and 3′ termini can be generated by various strategies and are linked to different selection constraints and biological roles (Shabalina et al, 2014), but we will elaborate furthermore on splicing of internal transcript sequences.

Splicing involves the stepwise actions of the spliceosome, a large and dynamic machine that at its core comprises a series of small nuclear ribonucleoprotein particles (snRNPs: U1, U2, U4/U6, and U5), each composed of small nuclear RNAs and their associated partners (Wilkinson et al, 2020). The small nuclear RNAs have both architectural roles to scaffold each snRNP and direct roles in recognizing splice sites; the protein components of U snRNPs also bind splice sites and processing motifs and execute RNA catalysis to achieve intron excision and exon ligation (Fig 1B). Altogether, the coordinated activities of RNA and protein components support the notion of the spliceosome as a “protein-orchestrated metalloribozyme” (Wan et al, 2020). Beyond this, snRNPs work in concert with an array of auxiliary splicing factors to specify correct splicing events.

Accurate splicing is critical because misprocessing causes inclusion or exclusion of sequences from the intended transcripts, and/or throws the downstream sequence out of frame. However, the selection of appropriate splice sites, even in a constitutive fashion, poses a conundrum. This is because of the limited primary sequence information for where to cleave and join 5′ and 3′ ends of introns, namely, at 5′ GU and AG-3′ dinucleotides (Fig 1B). Although minimal splice sites are embedded within longer motifs (e.g., 5′-GURAGU and YAG-3′), this is still insufficient to specify splicing because the spliceosome will encounter many more matches to such motifs that are productively spliced. This is especially an issue within long introns, which are prevalent in neural-expressed genes. Moreover, not all splice sites fully match these consensus motifs. Accordingly, the challenging process has been described as “finding splice sites within a wilderness of RNA” (Black, 1995).

When introns are short (<200 nt), the likelihood of illegitimate splice site matches is smaller, although certainly still well-documented in different kingdoms (Lim & Burge, 2001). In these settings, “intron definition” is sufficient to explain how a 5′ splice site can be cleaved and ligated to a downstream 3′ splice site (De Conti et al, 2013). This involves recognition of the 5′ splice site by U1 snRNP and of the 3′ splice site and upstream polypyrimidine tract by U2 snRNP and bridging of these subcomplexes to loop out the intervening sequence. However, when introns are long, it becomes less plausible that this strategy can explain splicing specificity. For example, some mammalian exons are flanked by megabase introns, which must harbor numerous illegitimate splice site matches. How can exons be located in this context? Here, the underlying mechanics of splicing itself are similar, but “exon definition” via interactions of 3′ splice site and downstream 5′ splice site complexes is thought to demarcate exon bounds, even when flanked by extremely long introns (Hollander et al, 2016). An exotic variant strategy involves recursive splicing, where sections of long introns are removed sequentially (Hatton et al, 1998; Duff et al, 2015; Sibley et al, 2015; Joseph et al, 2018).

**Figure 2.**
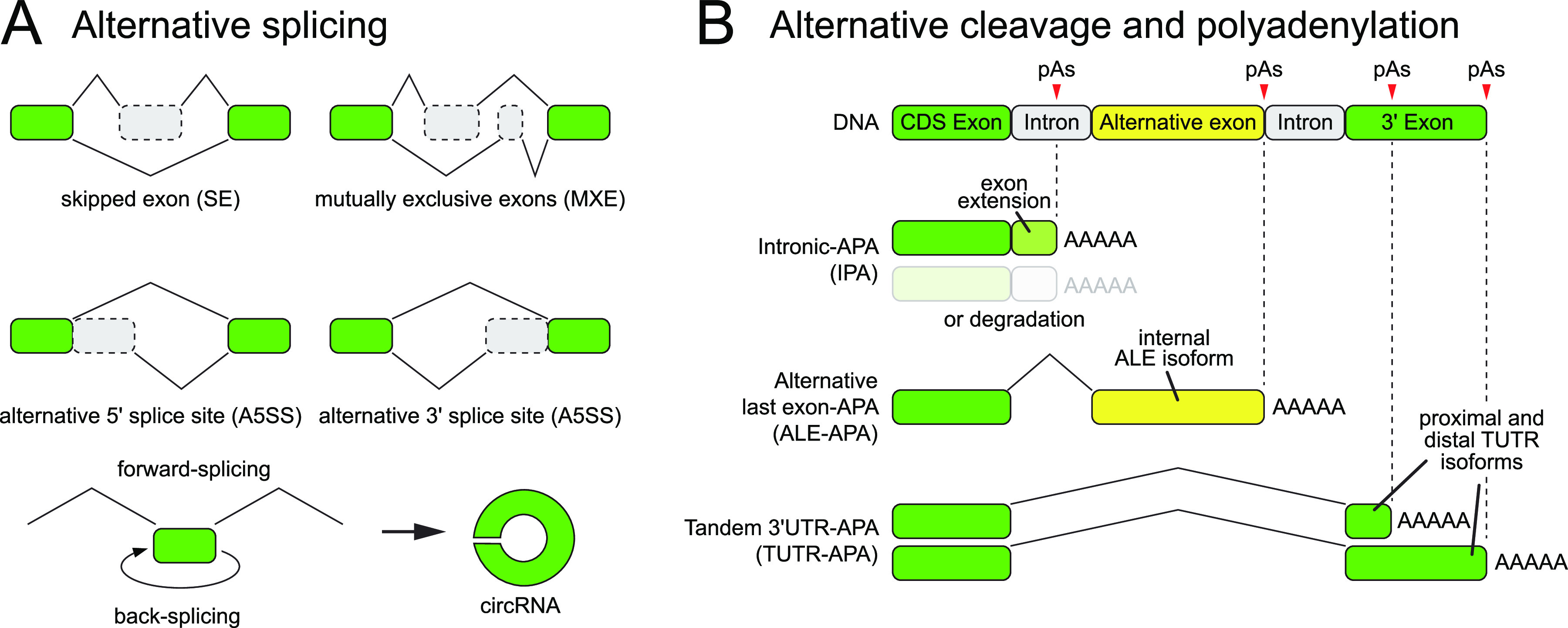
Classes of alternative splicing and alternative polyadenylation isoforms. **(A)** This schematic depicts classes of alternative transcripts that vary by inclusion or exclusion of internal exons. The major classes of alternative forward-spliced isoforms are depicted, and individual genes can be subject to multiple types of splicing regulation to yield highly complex isoform pools. Back-splicing can also occur, yielding circular RNA (circRNA) species. **(B)** Other splice isoforms can vary at 5′ ends due to alternative transcription start sites (not shown) or at 3′ ends (see panel [B]). **(B)** This schematic depicts classes of alternative transcripts that vary at their 3′ ends. By definition, cleavage and polyadenylation defines the 3′ end of a given transcript, but cleavage sites can be classified in different ways depending on their genomic location. Certain poly-A sites occur within the intron of a longer gene model, sometimes referred to as intronic polyadenylation. This can result in undesirable transcript truncation and might include spurious coding sequence, which together may trigger quality control pathways that degrade these faulty isoforms. In other cases, intronic polyadenylation generates a stable alternative last exon isoform that has a distinct function compared with the longer gene model. Finally, alternative polyadenylation within the same terminal exon can generate isoforms that differ only by 3′ UTR sequence.

Specific and accurate execution of constitutive exon ligation involves, in part, the active suppression of illegitimate, but biochemically valid, splice sites (Blazquez et al, 2018; Boehm et al, 2018; Joseph & Lai, 2021; Schlautmann et al, 2022; Taylor et al, 2022). However, the complexity of regulated, alternative splicing makes this even more of a conceptual challenge (Fig 2A). How are alternative splicing choices made, and in manners appropriate to cell-type and physiological state? In later sections, we will consider some of the mechanisms that implement alternative splicing.

### Fundamentals of 3′ end formation

Another fundamental aspect of eukaryotic mRNAs is that they bear terminal features that are not specified by the genome, that is, a 5′ cap and 3′ polyadenylate (pA) tail. These untemplated sequences protect mRNAs from degradation by a variety of professional exoribonucleases that destroy uncapped and/or untailed mRNAs. On the 3′ end, the cleavage and polyadenylation (CPA) machinery first identifies appropriate cleavage locations within primary transcripts, thereby separating the nascent RNA from RNA polymerase II, and subsequently adds the pA tail (Tian & Manley, 2017). These complex reactions are specified by primary sequence motifs (Fig 1C). The most critical of these is the polyadenylation sequence (PAS), typically AAUAAA and certain variants, located ∼20–30 nts upstream of the site of CPA (i.e., the pA site). The central factors in 3′ mRNA cleavage reside in the CPSF complex, which includes the CPSF30/WDR33 heterodimer that directly recognizes the PAS (Schonemann et al, 2014), and the CPSF73 endonuclease that cleaves nascent mRNA (Dominski et al, 2005; Mandel et al, 2006).

As with splicing, a similar puzzle exists as to how specificity of 3′ mRNA cleavage is achieved. The known PAS signals are themselves insufficient to explain accurate cleavage only at the 3′ ends of transcripts because 3′ UTRs and especially introns are enriched in AU sequences. Thus, numerous PAS-like sequences must seemingly be ignored by the CPA machinery to generate full-length mRNA. This specificity is explained in part by additional motif information located in the vicinity of bona fide 3′ cleavage sites, including upstream UGUA (recognized by the CFIm complex) and downstream U/GU (recognized by CstF complex) (Fig 1C). However, such motifs are not required to process many mRNAs, and thus have modulatory but not absolute roles in recognizing 3′ cleavage sites.

### ALE splicing and relation to intronic polyadenylation (IPA)

Because introns can be extremely long, they contain not only fortuitous splice site matches but also cryptic polyadenylation signals. Inappropriate action of the CPA machinery within an intron will create an alternative gene product with a distinct 3′ UTR from the downstream model, and high likelihood of encoding a truncated protein that may also bear a foreign C-terminus. One strategy to prevent this is “telescripting,” whereby U1 snRNP protects elongating Pol II from premature CPA (Berg et al, 2012; Almada et al, 2013). In particular, the need for U1-mediated suppression of early termination is especially overt within long introns (Oh et al, 2017), which preferentially exist in neural-expressed genes. This is also applicable to non-neuronal settings, as mammalian first introns are typically longer than downstream introns and require U1 telescripting (Kainov & Makeyev, 2020). Although U1 is most well-known for its role in splicing, it exists in a distinct complex with CPA factors to suppress cryptic PAS usage (So et al, 2019). More generally at terminal exons, U1 snRNP regulates 3′-end polyadenylation and gene expression via binding of its subunit U1-70K with the carboxy-terminal end of PAP (Gunderson et al, 1998; Fortes et al, 2003; Abad et al, 2008).

A single classification of IPA belies the complexity of functional outcomes on these alternative transcripts, and these can generate either 3′ terminal extensions of an existing exon or enable splicing into a distinct 3′ terminal exon (Fig 2B). In any case, IPA events are not formally intronic with respect to cognate alternative transcripts because CPA by definition generates the terminus of the respective exon (although it may be considered to be intronic with respect to a different gene model).

In some cases, fortuitous IPA events will generate a truncated transcript containing arbitrary sequence (Fig 2B). A typical outcome would be for such aberrant transcripts to be removed by quality control pathways, such as NMD. However, if production of the aberrant isoform is preferred because of sequence or genetic variation, this can substantially impede the production of the intended full-length product. However, IPA can also yield stable 3′ isoforms, which bear an internal ALE compared with isoforms carrying downstream 3′ sequence (Fig 2B). Although ALE isoforms can be classified within the rubric of alternative splicing, it is important to bear in mind that this can also reflect alternative CPA choice. In subsequent sections, we will discuss ALE splicing in the context of APA.

### Challenges for understanding alternative splicing and polyadenylation programs

Numerous laboratories and researchers are dedicated to unraveling the molecular strategies and mechanics of splicing and 3′ formation, simply in a constitutive fashion. However, the complexity of alternative mRNA splicing and 3′ formation events raises even more challenges. There is already seemingly not enough primary sequence information to distinguish “intended” processing events from the forest of “illegitimate” matches to splicing and polyadenylation signals. Given this, how can alternative splicing (AS, Fig 2A) and APA (Fig 2B) site usage be controlled appropriately across the genome, in cell-type, and condition-specific manners?

It is instructive to frame the breadth of these questions. If there were only a few AS and APA events, it might suffice to invoke specific regulatory phenomena at those loci. However, the advent of widespread genomic profiling has led to the realization that higher eukaryotes make extraordinarily broad use of both AS and APA. Indeed, available data from 15 yr ago revealed that the vast majority (>90%) of mammalian multi-exon genes undergo alternative splicing (Pan et al, 2008; Wang et al, 2008). This proportion continues to increase with ever deeper transcriptome profiling, comprising numerous subclasses (Fig 2A). Likewise, a most of the genes yield multiple 3′ isoforms in diverse metazoan species (Sandberg et al, 2008; Derti et al, 2012; Smibert et al, 2012). Again, these comprise numerous subclasses of APA isoforms (Fig 2B). Moreover, the collection of alternative isoforms from an individual gene can include many combinations of alternative internal exons and alternative 3′ terminal exons. These facts make it even more perplexing how such programs of isoform generation can be both accurate but also alternative to diversify the transcriptome.

### Diversity of neural gene expression and mRNA-processing programs

The long history of developmental biology studies has revealed a constellation of cell types that emerge from divisions of the fertilized egg, which must differentiate properly and assemble into distinct tissues and organs that underlie a functionally mature organism. The diversity of recognized cell types continues to increase with the application of technologies such as single-cell RNA sequencing (scRNA-seq). This technique promises to reveal “all” cell types and cell states, perhaps in the end revealing that every living cell is quantifiably a little different from every other. Although all researchers can debate what the most interesting cell types are, it is without question that neurons comprise one of the most diverse subclasses of cell types (Zeng, 2022). This can be appreciated even from perusing recent scRNA-seq catalogs. For example, the deep interrogation of the adult fly, which is much simpler than say the human, yielded ∼250 distinct high-quality cell types (Li et al, 2022). However, a deep dive into the development of the optic lobe, only a small portion of the nervous system, itself yielded ∼200 cell types (Ozel et al, 2021), whereas other individual tissues often resulted in tens of cell types. Thus, the nervous system exhibits extreme cellular diversity.

Diversity alone may suffice to explain the need for highly regulated gene expression in neurons. Many neural-specific genes, and accordingly neural-specific transcript isoforms, are required for distinct fates and/or maturation. Beyond this, neurons exhibit many unique properties that impose specialized regulatory requirements, beyond what exists in non-neural cell types. For example, neurons are famously the most polarized cell type of all and can be meters long in some animals. Accordingly, there must be strategies to transport transcripts to appropriate locations in neurons and for them to be subject to differential regulatory outcomes (such as altered stability or regionalized translation). Moreover, the unique physiology of neurons, coupled with their long lifespan encompassing the lifetime of the individual, likely underlie exceptional regulatory needs of neurons. Consistent with these notions, alternative splicing is especially prevalent in the nervous system (Raj & Blencowe, 2015; Vuong et al, 2016a; Gonatopoulos-Pournatzis & Blencowe, 2020), and neurons also express by far the longest 3′ UTRs of any cell type (Smibert et al, 2012; Miura et al, 2013; Agarwal et al, 2021; Lee et al, 2022). Moreover, within the neural population, there is extensive deployment of APA isoforms across neuronal compartments and in response to neural activity, with longer isoforms correlated with specific localization and altered stability (Tushev et al, 2018).

How are neural-specific programs of alternative isoforms implemented? Most evidence suggests that core spliceosome and CPA mechanics are similar across cell types, and certainly, numerous splice sites and pA sites are constitutive. Nevertheless, there are many reasons why these mRNA-processing machines may have differential activity on identical sequences in different cell types, such as cellular concentration of these complexes, the dynamics of RNA polymerase II, and especially, the influence of transacting factors. With respect to the latter, neurons might uniquely express factors that enable them to recognize mRNA-processing sites unavailable to other tissues, or alternatively, to suppress universally used sites. Both strategies can yield similar outcomes, and may work together.

### Neural RBPs that direct neural-specific splicing of internal exons

Although broadly expressed factors can clearly regulate cell-specific processes, RBPs that are specifically expressed or excluded from neurons comprise logical candidates for neural isoform regulators. Indeed, cell-specific RBPs that regulate neural splicing include members of the ELAV/Hu, Nova, Mbnl, Rbfox, Ptbp, CELF, and SRRM3/4 families, along with other factors such as TDP-43 (Raj & Blencowe, 2015; Vuong et al, 2016a). These factors were classified based on functional evidence that their loss-of-function abrogates neural isoform programs, and in many cases their gain-of-function is sufficient to induce such isoforms. Such effects are studied at individual loci, using RT-PCR to test alternative splicing at individual exons or endogenous genes or within minigene reporters, and at the genome-wide level using RNA-seq data to quantify alternative splice junctions under different genetic conditions.

**Figure 3.**
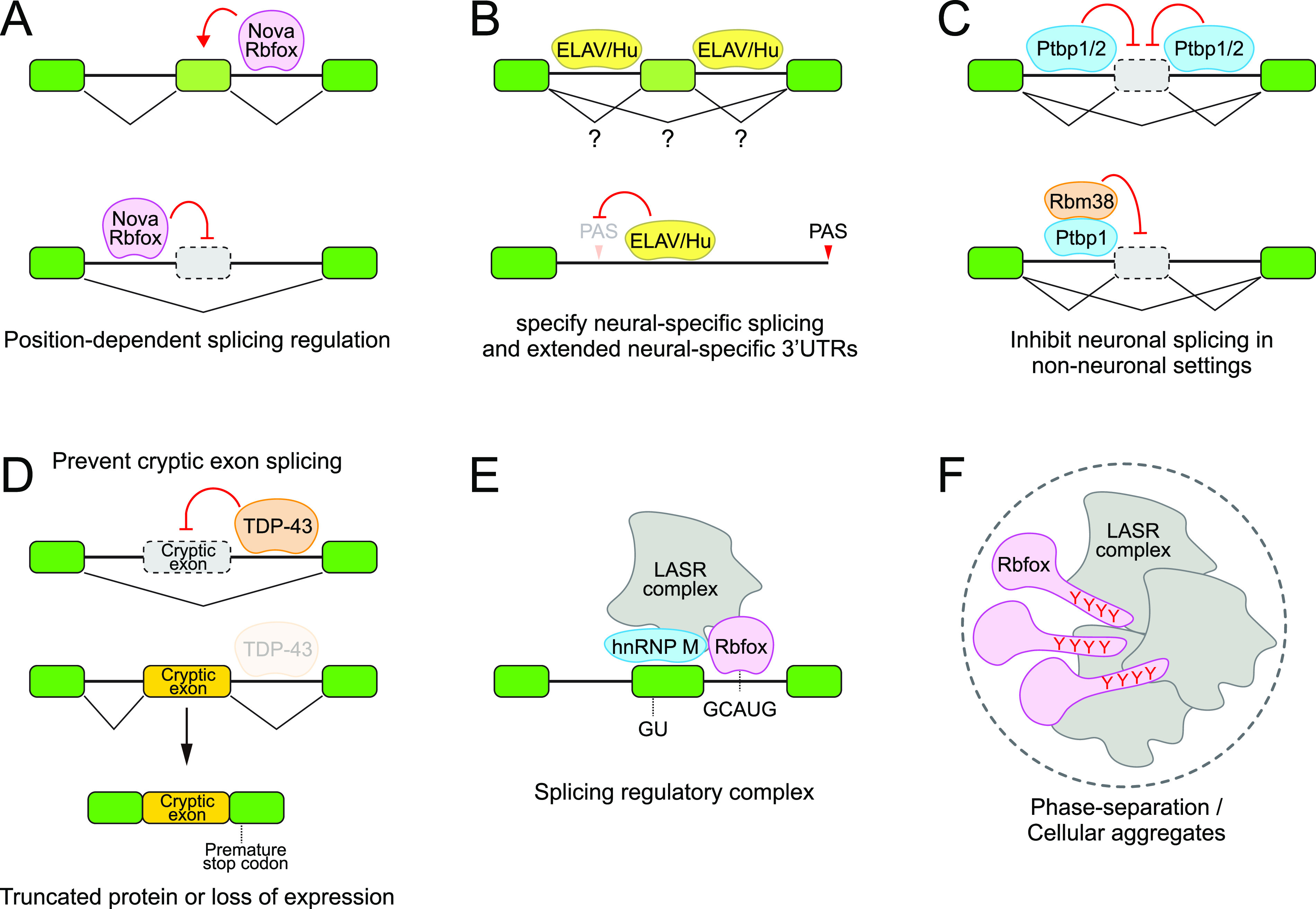
Mechanisms of RNA-binding proteins (RBPs) that specify neural alternative splicing. **(A)** Nova and Rbfox are examples of neural RBPs with position-specific influence on whether they promote or inhibit exon inclusion. **(B)** ELAV/Hu RBPs are examples of multifunctional neural isoform regulators that can direct both internal exon inclusion and exclusion and 3′ UTR extension. **(C)** Neural isoform programs can be directed by neural-restricted RBPs, but reciprocally, Ptbp1/2 are examples of RBPs that globally inhibit neuronal splicing in non-neuronal settings. **(D)** Some RBPs prevent inappropriate, “cryptic” splicing events, which may be of particular significance in neurons due to frequent long-gene models. **(E)** Although these diagrams highlight simple interactions for isoform control, RBPs do not necessarily work in isolation. **(A, F)** Rbfox (from panel [A]) operates within a large regulatory complex termed “LASR,” which itself may exist within large multivalent, phase-separated domains (F).

Although genetic data by itself does not rule out indirect effects, many of these RBPs bind specifically in the vicinity of alternative exons, often within flanking intronic regions, suggesting they directly guide isoform selection. However, manipulation of many of these factors can alter both exon inclusion and exclusion events. Can the same factor achieve opposite mRNA-processing outcomes? Although rules are not absolute, the positional binding of RBP-splicing regulators can be correlated to functional outcome. These principles were revealed by computational prediction of conserved RBP motifs and with cross-linking and immunoprecipitation (CLIP) maps of RBP occupancy. For example, with both Nova (Ule et al, 2006) and Rbfox (Underwood et al, 2005; Zhang et al, 2008), intronic binding downstream of a target exon promotes inclusion, whereas upstream intronic (and/or exonic binding) preferentially induces skipping of the adjacent exon (Fig 3A).

Artificial tethering assays, in which an RBP is recruited to a target transcript independently of its RNA-binding domain, can bolster the evidence for direct and position-dependent effects on splicing. For example, recruitment of Rbfox1 to the downstream intron can promote exon inclusion, whereas tethering to the upstream intron promotes skipping of the adjacent exon (Sun et al, 2012). Such assays can be refined by tethering specific portions of the splicing regulator. An especially informative outcome is when domains outside of the RNA-binding domain(s) are sufficient to alter target splicing, which would indicate a modular “effector” domain, and also provide evidence that RBP occupancy does not alter mRNA processing solely through steric or competitive interactions. In the case of Rbfox1, its C-terminus is sufficient to mediate both exon inclusion and exclusion, although the domain requirements are not identical (Sun et al, 2012). As another example, the N-terminal region of PTB, classically thought to be a splicing repressor, can activate splicing when tethered directly downstream of a target exon (Llorian et al, 2010). Finally, the density of binding sites at a target locus may be relevant. The ELAV/Hu family of RBPs also mediate broad programs of neural alternative splicing in mammals (Ince-Dunn et al, 2012; Scheckel et al, 2016) and *Drosophila* (Carrasco et al, 2020; Lee et al, 2021), including both exon inclusion and exclusion events (Fig 3B). Both regulatory outcomes are associated with local binding of ELAV/Hu RBPs, but this does not clearly indicate regulatory outcome. It may be that the local density of upstream intronic-binding site/events is higher when the target exon is suppressed, as opposed to included (Ince-Dunn et al, 2012; Lee et al, 2021).

It is relevant to bear in mind two sides of the coin. The neural-specific isoform program must not only be implemented using positive regulators but also using negative regulators. For example, Ptbp1/2 is global repressors of neural-specific alternative splicing (Fig 3C). They have distinct expression, with Ptbp1 expressed broadly outside of the nervous system and in neural stem cells, whereas Ptbp2 is restricted to immature neurons. However, neither protein is present in mature neurons. Ptbp1/2 binds similar CU-rich consensus sites, often within upstream intron polypyrimidine tracts, and occupies many of the same target genes (Keppetipola et al, 2012). Genetic tests indicate that their combined action prevents the usage of alternative splicing events that should be restricted to functional neurons (Ashiya & Grabowski, 1997; Chan & Black, 1997; Boutz et al, 2007; Spellman et al, 2007). Beyond roles in blocking neuronal exon inclusion, PTBP1 also promotes splicing of certain non-neuronal exons, which involves position-dependent binding (Hamid & Makeyev, 2017; Iannone et al, 2023).

Other factors protect the neural transcriptome from aberrant/cryptic-splicing events. An unexpected strategy comes from studies of the nuclear RBP TDP-43, whose cytoplasmic aggregation or loss is a hallmark of the neurodegenerative syndromes frontotemporal dementia and amyotrophic lateral sclerosis (Neumann et al, 2006). TDP-43 is preferentially expressed in the nervous system and was later demonstrated to have a major role in mRNA processing. In particular, loss of TDP-43 results in inclusion of hundreds of non-conserved, cryptic exons (Fig 3D), which can prevent translation of normal proteins and/or induce nonsense-mediated decay (Ling et al, 2015). Although it is evident how broad mis-splicing of neural genes could generally be deleterious, some individual critical misregulated targets have emerged, such as *stathmin2* (Klim et al, 2019; Melamed et al, 2019; Baughn et al, 2023) and *Unc13a* (Brown et al, 2022; Ma et al, 2022). Therefore, a critical aspect of maintaining the fidelity of neural isoforms is to prevent inappropriate splicing choices.

To broaden the catalog of splicing regulators, forward genetic screening offers an unbiased perspective (Albulescu et al, 2012; Han et al, 2017). Recently, systematic insights were gained from multiplexed, deep sequencing of RT–PCR amplicons, testing 108 neural alternative splicing events against 1,416 knockdown perturbations in mouse Neuro-2a (N2A) cells (Han et al, 2022). These targets were selected from a comprehensive set of neural AS events and were nominated to be emblematic for different regulatory paradigms across different brain regions. With quantitative and large-scale measurements, about half of the perturbations altered AS, including positive and negative factors. These hit all the known neural splicing regulators and also provided leads into new mechanisms.

Curiously, many ubiquitous RBPs can mediate cell-specific isoform regulation. For example, the broadly expressed RBPs Puf60 and Rbm38 were preferentially required for specific neural AS programs, with Puf60 correlated with SRRM4 and SRSF11, and Rbm38 exhibiting similarity to Ptbp1 (Han et al, 2022). Rbm38 was perhaps even less likely as a cell-specific splicing regulator because it is a multifunctional regulator of both nuclear and cytoplasmic events, but not previously known as a neural splicing regulator. Interestingly, Rbm38 is generally expressed at low levels in neural tissues compared with other organs and inhibits many neural AS events. It may do so through a combination of strategies, in part having indirect effects by post-transcriptionally regulating other relevant RBPs. However, it also physically interacts with many neural-restricted AS regulators, including Ptbp1, and a portion of Rbm38-mediated splicing events are dependent on Ptbp1/2. On the other hand, Puf60 emerged as a positive regulator of specific neural AS events. In this case, this involves tissue-specific AS of Puf60 itself because brain isoforms include an AS exon that affects its protein partners. Importantly, not only Rbm38 and Puf60 are required for neural-specific splicing, embryonic stem cells that lack Rbm38, or that constitutively express or lack *Puf60*-exon5, exhibit multiple defects across the path of directed differentiation into neurons (Han et al, 2022).

Beyond these examples, there are undoubtedly other regulators of neural AS whose stories await. Moreover, the screening strategies used can be applied to further sets of AS events and also extended to other cell systems. Ultimately, such functional assays for neuronal splicing may be most powerful if applied to genuine neurons.

### Combinations and higher order complexes of RBPs regulate neural isoform choices

Many studies characterize manipulations of an individual factor, which can demonstrate their necessity, and in some cases sufficiency, to induce broad isoform changes. Still, this does not mean that they work in isolation. Many laboratories independently encounter the same RBPs in the context of neural mRNA processing, suggesting that combinations of transacting RBPs may act at the same exons, either working cooperatively or in opposition. Beyond target co-regulation by paralogs, different RBPs can sometimes have similar functional effects. For example, as mentioned, Nova and Rbfox RBPs both promote neuronal isoform programs and exhibit similar positional binding, that is, distinct for inclusion versus exclusion targets. About 15% of Nova direct targets bear highly conserved sites for Rbfox, and functional tests validate that splicing of some targets is co-regulated by both factors, in the same direction (Zhang et al, 2010). However, the influence of Nova and Rbfox at some targets is antagonistic. In other contexts, functional interplay of Celf and Rbfox (in T cells) (Gazzara et al, 2017), and of Celf and Mbnl (during developmental transition in heart development) (Kalsotra et al, 2008) control programs of alternative splicing. In contrast, Ptbp1/2 is mostly considered as inhibitors of neural splicing, thereby excluding neural isoforms from non-neural settings. Accordingly, many of these neural splicing factors counteract Ptbp1/2 activity to install neural isoform choice. Overall, temporal waves, cell-type–specific expression, and combinatorial action of neural-splicing regulators such as Nova, Rbfox, Mbnl, and Ptbp families, seem to underlie dynamic programs of alternative splicing during brain development (Weyn-Vanhentenryck et al, 2018).

Ultimately though, these RBP-splicing regulators cannot only influence each other, they must somehow impinge on the spliceosome. However, it remains the case that our knowledge is largely correlative. For example, an RBP may exhibit global enrichment near regulated exons, and may physically associate with spliceosome components. However, neither this necessarily mean that all targets whose splicing changes upon manipulating the RBP are directly bound or regulated nor does it mean that interaction with a given spliceosome factor is central to regulation. Therefore, it becomes relevant to know if RBP-splicing regulators mediate their action through larger complexes.

This scenario was demonstrated with Rbfox proteins, which bind the well-defined motif (U)GCAUG. Strikingly, CLIP assays of Rbfox in the soluble nuclear fraction showed preferred association with 3′ UTRs, but CLIP data from the high molecular weight nuclear fraction showed its predominant association within introns in the vicinity of regulated exons, and overlapping consensus sites (Damianov et al, 2016). This suggested that conventional CLIP was unable to reveal the broad impact of Rbfox on co-transcriptional processing because it resides in an insoluble fraction, that is, normally discarded. In fact, nuclear Rbfox proteins primarily reside in an insoluble, large assembly of splicing regulators (LASR, Fig 3E), which includes a variety of other splicing factors (hnRNP-M, hnRNP-H, hnRNP-C, Matrin3, NF110, NF45, and DDX5) in similar frequency to Rbfox (Damianov et al, 2016). Moreover, Rbfox could repress splicing via the GU-rich binding site for the LASR component hnRNP-M. Overall, these studies indicate that a given splicing regulator does not necessarily work in isolation or does it necessarily require strict matches to its consensus for target selection.

Recently, the LASR complex was extended to include the Rett syndrome factor methyl-CpG–binding protein 2 (MeCP2), with evidence that MeCP2 disease mutants impair recruitment of Rbfox to LASR accompanied by defective splicing of Rbfox targets (Jiang et al, 2021). In addition, MeCP2 disease variants also disrupted the phase-separated condensates that normally contain Rbfox and MeCP2. More generally, the partitioning of cellular proteins via liquid–liquid phase separation has broadly captured the imagination of the scientific community as a means of compartmentalizing and enhancing cellular reactions (Shin & Brangwynne, 2017), and diverse RNA-related processes have been linked to liquid–liquid phase separation (Han et al, 2012; Kato et al, 2012; Roden & Gladfelter, 2021; Wiedner & Giudice, 2021). In fact, some of these neural-splicing regulators have also been shown to be involved in cellular aggregates. For example, the C-terminal, disordered, tyrosine-rich domain of Rbfox was shown to mediate cellular aggregates with LASR components (Fig 3F), which mediates exon inclusion (Ying et al, 2017).

These seem to embody general principles. First, alternative splicing of other splicing factors (hnRNP-A, -D, and -D–like) serves to include exons that encode disordered, tyrosine-rich regions that mediate multivalent assemblies (Gueroussov et al, 2017; Batlle et al, 2020). Interestingly, many of these exons were acquired recently in the mammalian lineage and were proposed to enhance alternative splicing diversity in mammals, and some of these exons are disease targets. Second, other factors that influence neural splicing and are connected to neurological disease (e.g., Fus, TDP-43, hnRNPA1, and hnRNPA2B1) also participate in phase separation (Mann & Donnelly, 2021). Although there are justifiable concerns on how to determine casual in vivo roles of phase separation on cellular processes (Musacchio, 2022), it is certain that more will be forthcoming in this area.

### Microexon regulation in neurons

A specialized type of neural-preferred splicing concerns microexons (Ustianenko et al, 2017; Gonatopoulos-Pournatzis & Blencowe, 2020). Operationally defined as being smaller than typical short exon sizes (∼50 nt), microexons are easily overlooked and may not be handled properly by default mapping of RNA-seq data. However, careful revisitation of deep RNA-seq data indicates there are >1,000 microexons shorter than 25 nt in mammalian data, with the shortest comprising only 3 nt (Irimia et al, 2014; Li et al, 2015). Although they encode only a few amino acids, microexons are often highly conserved and preserve reading frame. Thus, we can infer that this class is under selection as functional protein variants. Although these frequently lack sufficient content to encode alternate protein domains, as can occur with typical cassette exon isoform variants (Yang et al, 2016), microexon insertions could modulate existing protein domains, provide novel sites of protein–protein interaction, and/or include protein modification sites. By contrast, other microexons specifically contain nonsense codons (i.e., “poison exons”), and presumably comprise a negative regulatory strategy. One prominent example is that numerous splicing factors are controlled by poison exons (Lareau et al, 2007; Leclair et al, 2020), indicating feedback regulation, and poison exons are notably used in the nervous system (Carvill & Mefford, 2020).

Microexons present special mechanistic challenges for splicing, as they verge on the limits of concurrent occupation of 3′ and 5′ splicing complexes for exon definition. Their small size also presents limited sequence space to encode desired amino acids and exonic splicing enhancers to promote their inclusion. However, a potential clue as to the regulation of microexons comes from their preferred inclusion in neurons, either as constitutive exons of neural-specific genes or as alternatively spliced neuron isoforms (Irimia et al, 2014; Li et al, 2015). This suggests that intrinsic features of their layout and/or neuronal-specific factors might enable or stimulate microexon inclusion.

**Figure 4.**
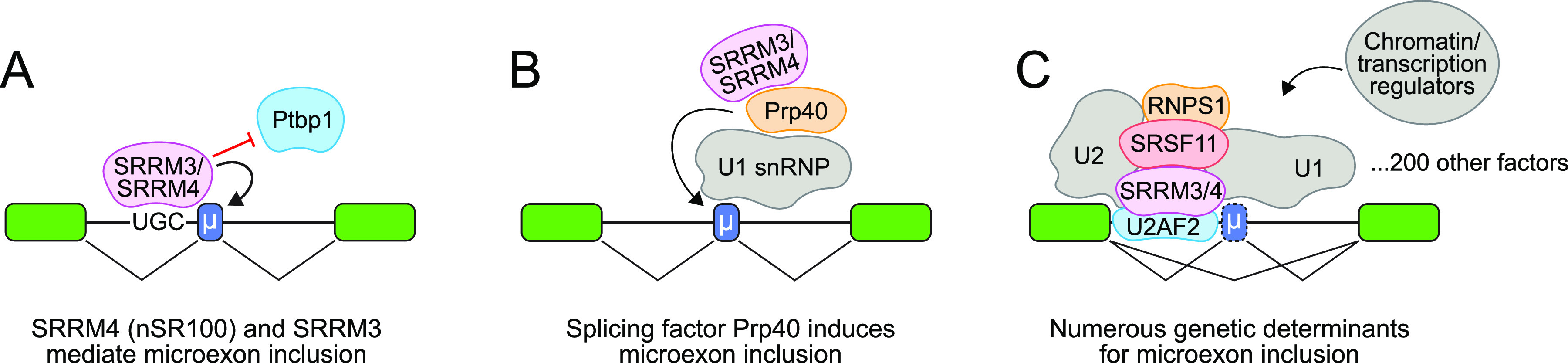
Mechanisms for neural microexon inclusion. The small size, suboptimal features, and predominant inclusion of microexons in neurons, all suggest the existence of specific regulatory pathways for their recognition. **(A)** Paralogous mammalian factors SRRM4 (nSR100) and SRRM3 (functionally equivalent to *Drosophila* SRRM234) recognize UGC motifs upstream of microexons to promote their inclusion, which is antagonized by Ptbp1. **(B)** The core splicing factor Prp40 is involved in neuron-specific microexon inclusion. **(C)** Many other factors, as well as chromatin and transcriptional features, are coordinately involved in microexon inclusion.

Indeed, several neural-splicing regulators mentioned above, including Rbfox, PTBP1-2, and nSR100/SRRM4, are also involved in neural microexon splicing (Fig 4A) (Irimia et al, 2014; Li et al, 2015). For example, broadly expressed PTBP1 represses the inclusion of neuronal microexons that are promoted by Rbfox (Li et al, 2015). Interestingly, nSR100/SRRM4 has selectivity for neural microexon splicing. It was earlier identified as a neural-specific SR-related protein that is required for neural differentiation and is both necessary and sufficient for a global program of neural exon inclusion (Calarco et al, 2009; Nakano et al, 2012; Raj et al, 2014). However, it was later appreciated that knockout mice preferentially lose the microexon class of alternative splicing events (Quesnel-Vallieres et al, 2015). Moreover, its paralog SRRM3 also controls microexon splicing (Torres-Mendez et al, 2019), particularly in locations where SRRM4 is not expressed (Ciampi et al, 2022).

How does SRRM3/4 achieve specificity? It turns out that their target exons generally have weaker 3′ splice sites with weaker polypyrimidine (U/C) tracts, and also bear PTBP-binding sites, which along with their unconventional length makes them prone to skipping (Fig 4A). Interestingly, one of the features of weak polypyrimidine tracts is the presence of guanosine, with enrichment of UGC motifs. These UGC motifs are bound by nSR100/SRRM4, and in vitro splicing assays show that nSR100/SRRM4 can counteract PTBP1-mediated repression to promote microexon inclusion (Raj et al, 2014). Overall, the logic appears to be that neural microexons are relatively weak in their upstream 3′ splice sites but are selectively bound by nSR100/SRRM4 (and presumably SRRM3) in neurons to enable their cell-specific inclusion.

Interestingly, the U1 spliceosome component Prp40 is also selectively required for neuronal microexon inclusion in *Caenorhabditis elegans* and mammals (Choudhary et al, 2021), revealing an unexpected role for a core splicing factor in alternative splicing (Fig 4B). It was proposed that Prp40 aids intron definition to process microexons, for which exon definition is sterically challenged. Moreover, this suggests that transacting factors could in some way impinge upon Prp40 to selectively modulate microexon splicing. This notion is supported by the fact that several RBPs that promote microexon inclusion exhibit physical association with Prp40, including with nSR100/SRRM4 in mammals (Gonatopoulos-Pournatzis et al, 2018; Torres-Mendez et al, 2019) and with ELAV (exc-7) in *C. elegans* (Choudhary et al, 2021).

The possibility of additional microexon regulators was opened by CRISPR screening (Gonatopoulos-Pournatzis et al, 2018). These efforts used a clever dual fluorescence reporter containing a +1 reading frame microexon upstream of GFP and +1mCherry. Skipping of the microexon permits GFP translation, but inclusion of the microexon places GFP out of frame, but now enables mCherry translation. Expression of this reporter in Neuro 2A (N2A) cells yields double-positive cells, indicating production of both microexon skipped and included isoforms. However, these can be biased into GFP-only or mCherry-only cell populations upon loss and gain of SRRM4, respectively. Broadening this to whole-genome screening identified ∼200 genes that modulate neuronal microexon inclusion. The top hits included known factors such as SRRM4, SRRM3, and Rbfox2. Curiously, not all RBP hits are neural-specific factors, but instead include many general mRNA-processing factors, mRNA metabolism, and other broadly expressed RBPs. Among the latter, SRSF11 and RNPS1 were shown to coactivate microexon inclusion, in concert with neuronal SRRM4 (Fig 4C). Other broad classes of factors included chromatin and transcriptional regulation, potentially providing links to co-transcriptional processing or promoting expression of regulators of “core” microexon regulators.

Of note, microexon regulators are biased for misregulation in autism (Quesnel-Vallieres et al, 2016; Gonatopoulos-Pournatzis et al, 2018, 2020a). Thus, settings of neural dysfunction may be associated with specialized disruption of neuronal isoform programs, such as microexons. Indeed, genetic loss of enhancer of microexons (eMIC) domain from *Drosophila* SRRM234 selectively impairs microexon inclusion and causes defects in neural excitability and behavior (Torres-Mendez et al, 2022). Further studies will address and elaborate on how microexon splicing might control human neural differentiation and function in normal and disease situations.

### Cross-regulation between RBP paralogs during neural splicing regulation

As many splicing regulators belong to families, this can complicate functional identification of RBPs that direct neural isoform diversity. In particular, compensation by other family members may cause individual knockouts to not score as an initial hit, or at the least, to strongly underestimate the regulatory network of a family. Indeed, compound knockouts of an RBP family often yield stronger phenotypes and stronger molecular defects. Indeed, alterations in neural splicing programs were enhanced in double mutants of ELAV/Hu members *HuC/D* in mice (Ince-Dunn et al, 2012) and with *Mbnl1/2* double-knockout mice (Goodwin et al, 2015), relative to respective single mutants. However, rarity and lethality of compound mutants can pose difficulties for animal genetics of these important regulators. In this case, appropriate cell systems and CRISPR engineering can also provide invaluable models. For example, embryonic stem cells can be differentiated into numerous types of defined neurons, and this enabled the study of broad splicing defects in *Rbfox1/2/3* triple-mutant ventral spinal cord neurons (Jacko et al, 2018). In these cases, many hundreds of aberrant neural-splicing events (both exon inclusion and exclusion) can be detected in compound mutants of an RBP family.

**Figure 5.**
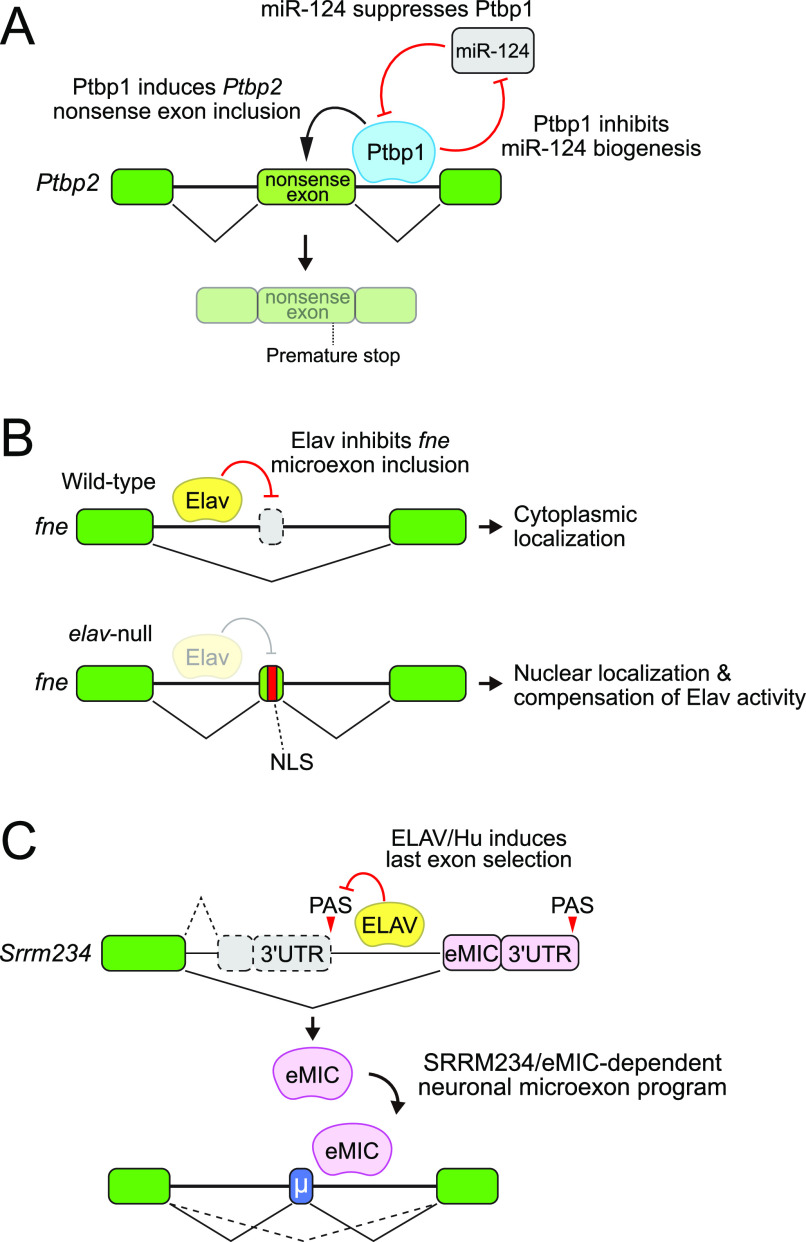
Hierarchical control of neural splicing. **(A)** Sequential functions of the mammalian paralogs Ptbp1 and Ptbp2 are mediated by their cross-regulation and by miRNA control. Ptbp1 induces the inclusion of a nonsense-bearing exon in *Ptbp2*. In neurons, the induction of neural-specific miR-124 down-regulates *Ptbp1*, facilitating the transition to Ptbp2 function. Reciprocally, Ptbp2 also represses miR-124 biogenesis. **(B)** Sequential functions of the *Drosophila* paralogs Elav and Fne, members of the ELAV/Hu family, are mediated by their cross-regulation. In normal neurons, Elav suppresses inclusion of an internal *fne* microexon that confers nuclear localization of the normally cytoplasmic Fne protein. In Elav-low neurons (e.g., in *elav* mutants), the inclusion of the Fne microexon facilitates its nuclear activities in mRNA processing to induce neural splicing and 3′ UTRs. **(C)** *Drosophila* SRRM234 expresses a distal neural-specific ALE isoform that contains the eMIC domain, which mediates microexon inclusion. Distal ALE splicing of *Srrm234* is induced by neural ELAV/Hu family factors (i.e., Elav and Fne), and the SRRM234-eMIC isoform subsequently directs the global neural microexon program.

However, there can be complex relationships among family members, and paralogs may not necessarily function together in time or in space. The Ptbp family nicely illustrates a temporary hierarchy. As mentioned, Ptbp1 is expressed in neural stem cells and in non-neural cells but is down-regulated in post-mitotic neurons, concomitant with induction of Ptbp2; mature neurons eventually lose Ptbp2 (Keppetipola et al, 2012). Knockout mice and cells demonstrate independent requirements for ubiquitous and neural PTBP members (Licatalosi et al, 2012; Linares et al, 2015; Vuong et al, 2016b). Their distinct expression is not only due to transcriptional regulation but also by multiple post-transcriptional regulatory strategies (Fig 5A). First, Ptbp1 forces inclusion of a nonsense Ptbp2 exon so that Ptbp2 is non-functional when both genes are transcribed (Boutz et al, 2007; Spellman et al, 2007). In addition, Ptbp1 is suppressed post-transcriptionally by neuron-specific miR-124 (Makeyev et al, 2007). A final twist is that the biogenesis of miR-124 is post-transcriptionally blocked by PTBP1 (Yeom et al, 2018). Therefore, a series of interconnected cross-regulatory interactions implements mutually exclusive expression of Ptbp1/2 during the neural lineage (Fig 5A).

A different type of cross-regulatory splicing regulation occurs with *Drosophila* ELAV/Hu members *elav* and *fne*, which are global regulators of the neural isoform landscape (Carrasco et al, 2020; Wei et al, 2020; Lee et al, 2021). ELAV/Hu family members are expressed pan-neuronally but exhibit temporally distinct expression, with Elav expressed first, followed by Fne and Rbp9 (Zaharieva et al, 2015). Until recently, there was not a compelling reason to consider Fne (or Rbp9) as regulators of mRNA processing because they dominantly accumulate in the cytoplasm and presumably regulate gene expression in this compartment (Zaharieva et al, 2015; Alizzi et al, 2020). Moreover, *fne* mutants are mostly normal although they exhibit certain dendrite morphogenesis defects (Alizzi et al, 2020), whereas *elav* mutants are lethal during late embryogenesis and early larvae. However, in *elav* mutant neurons, *fne* undergoes a splicing switch to include a previously unannotated exon near its hinge region (Fig 5B). The Elav-repressed isoform of Fne preferentially localizes to the nucleus, suggesting that it provides a functional backup that is hierarchically suppressed by Elav (Carrasco et al, 2020; Wei et al, 2020). *elav* mutants exhibit selective changes in neural splicing (including of *fne*), whereas *fne* mutants are relatively normal; however, the *elav/fne* double-mutant deregulates nearly 1,000 neural alternative splicing events (Carrasco et al, 2020; Lee et al, 2021).

To what extent do RBP paralogs harbor intrinsically distinct functions? Or, could their separable genetic requirements be explained simply by differential expression? In the case of Ptbp1/2, some exons exhibit differential sensitivity in selected assays (Vuong et al, 2016b). However, a more rigorous genetic test was performed by asking to what extent conditional knockout of *Ptbp2* could be rescued by concomitant activation of a *Ptbp1* knockin allele. Although the exogenous *Ptbp1* allele lacks some of its endogenous regulatory control, it could phenotypically rescue certain *Ptbp2–cKO* brain defects, but not others (Vuong et al, 2016b). In addition, side-by-side CLIP maps showed that Ptbp1/2 exhibit highly similar target occupancy, even for cases where the exon in question was sensitive to only one of the factors. Therefore, it was inferred that other determinants, such as differential binding partners, may influence splicing outcome. In the case of Elav/Fne in *Drosophila*, precise engineering was performed at the endogenous loci. Genetic analysis of an *fne* allele that forces usage of its nuclear isoform can rescue the lethality of *elav* hypomorph mutants, and the introduction of the nuclear *fne* isoform at the endogenous *elav* locus supports full phenotypic rescue (Carrasco et al, 2022). Thus, the functional hierarchy of these factors is enforced both by cross-regulation of *fne* alternative splicing by Elav, as well by temporal sequence (Elav is expressed first and thus has a dominant requirement).

Finally, recent work elaborated hierarchical activity of different neural-splicing factors. Mammalian SRRM4 and SRRM3 contain a C-terminal eMIC domain, which is lacking from the ancestral splicing factor SRm300/SRRM2. Surprisingly, the eMIC domain is present in the single *Drosophila* ortholog SRRM234 but is only expressed in neural isoforms as a consequence of ALE splicing (Torres-Mendez et al, 2019). Evolutionary tracing demonstrates that the ancestral bilaterian state is for multiple distinct isoforms to be encoded by an individual *SRRM234* gene, whose neural distal ALE splice isoform selectively promotes neural microexon processing. In fact, ELAV/Hu RBPs are required to induce the neural eMIC-containing SRRM234 isoform, which subsequently executes neural microexon splicing (Fig 5C) (Torres-Mendez et al, 2022). Therefore, a hierarchy of splicing regulators relay different neural isoform programs in series.

### Control of neural-specific 3′ UTR extensions by ELAV/Hu RBPs

Moving to isoforms regulated by APA, neurons are again particularly notable as they express hundreds of extended 3′ UTR isoforms that are absent from other cell types, in both vertebrates and invertebrates (Hilgers et al, 2011; Shepard et al, 2011; Smibert et al, 2012; Ulitsky et al, 2012; Lianoglou et al, 2013; Miura et al, 2013). Some extended neural 3′ UTRs are ∼20 kb, making them some of the longest annotated exons known. Importantly, some of the longest 3′ UTRs were validated as dominant transcript isoforms using Northern blotting, thereby ruling out the possibility that these might join discontinuous regions and/or reflect minor species (Smibert et al, 2012; Miura et al, 2013). In general, the identity of regulatory factors that confer numerous cell-specific 3′ landscapes were unknown, and the breadth of such distinct 3′ programs has only broadened with the use of scRNA-seq (Agarwal et al, 2021; Lee et al, 2022). Manipulation of several RBPs that affect neural splicing can also alter neural APA isoforms, including Nova2 (Ule et al, 2006), Fus (Masuda et al, 2015), and MBNL family proteins (Batra et al, 2014). However, as many of these do not exhibit strong directional effects on 3′ UTR isoforms, it remained unclear if they are central determinants of the global extended neuronal 3′ UTR landscape. Alternatively, other general features, such as Pol II speed, the concentration of core CPA factors, or the relative strength of proximal APA sites, might contribute to this process (Miura et al, 2014). Still, precedent literature reported that mammalian PTB (Castelo-Branco et al, 2004; Hamon et al, 2004; Le Sommer et al, 2005) and HuR (Dai et al, 2012) can repress CPA. These seem to occur by impairing CstF recruitment, possibly by competing for CstF binding to the downstream sequence element.

**Figure 6.**
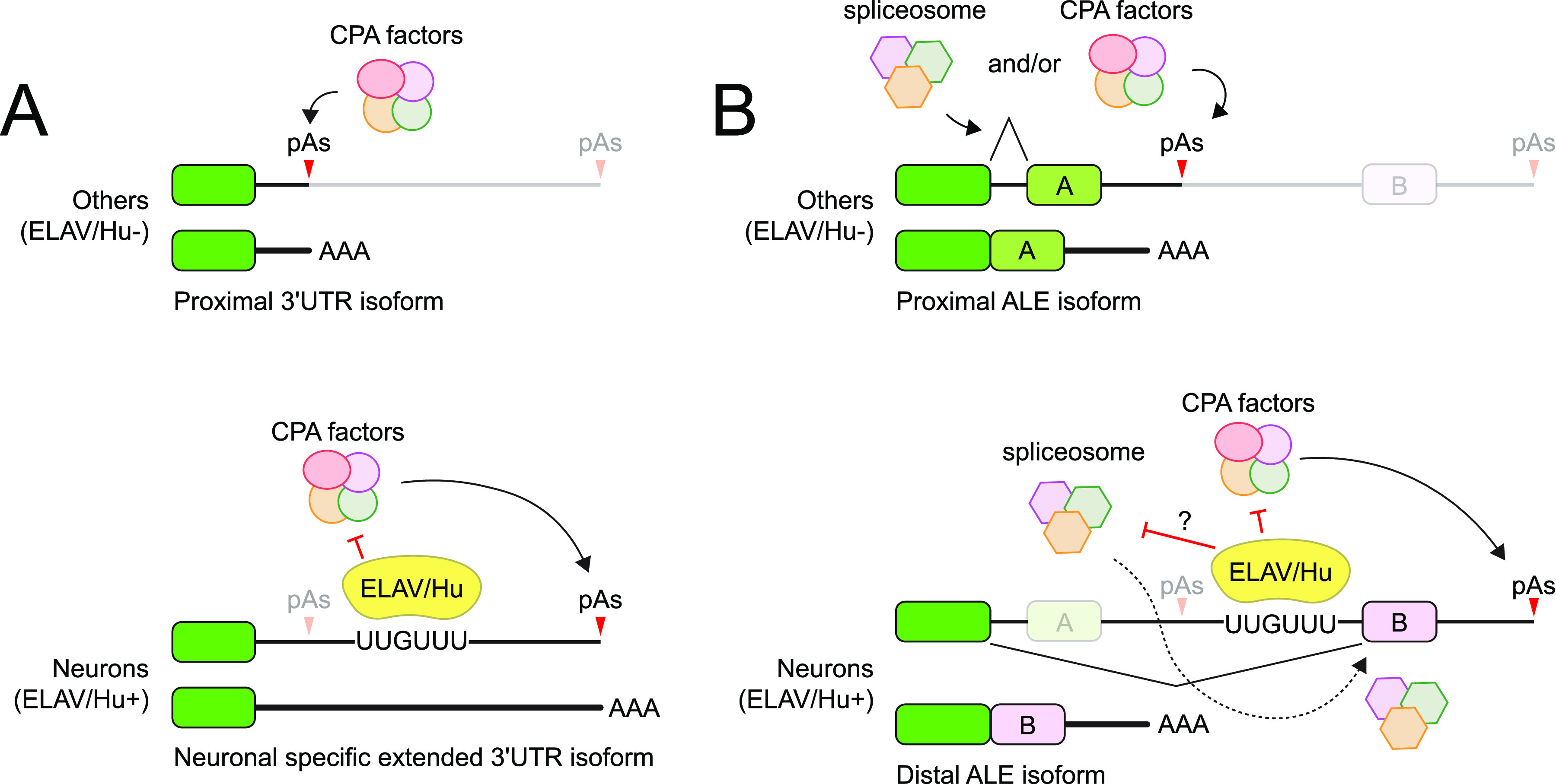
Control of distal alternative last exon (ALE) inclusion and terminal 3′ UTR lengthening by ELAV/Hu RBPs. ELAV/Hu RBPs (including *Drosophila* Elav and Fne) mediate global programs of 3′ UTR extension and switching from proximal to distal ALE isoforms in neurons. Both of these can be considered to be mediated by suppression of proximal polyadenylation signal (PAS) usage. **(A)** In non-neural cells, the CPA machinery effectively uses proximal PAS to generate short 3′ UTR isoforms. In neurons, ELAV/Hu RBPs bind downstream of PAS to locally inhibit CPA, which can instead cleave the extended transcript at a downstream site to yield the neural-extended 3′ UTR isoform. **(B)** ALE splicing may in principle involve either the spliceosome or the CPA machinery. However, ELAV/Hu factors have an analogous, broad role, to suppress the usage of intronic PAS that generate proximal ALE isoforms. This permits Pol II to transcribe further downstream, making the distal ALE isoform available for splicing. It is unclear if ELAV/Hu factors impact spliceosome function during ALE splicing, although this RBP class is also a global regulator of internal splice isoforms in neurons.

Only recently it was determined that the family of neuronal ELAV/Hu RBPs globally instructs the generation of neural-extended 3′ UTRs in *Drosophila* (Carrasco et al, 2020; Wei et al, 2020). These studies built on foundational work that Elav inhibits internal 3′ usage at the *erect wing* gene to permit expression of a downstream, neural-specific, ALE isoform (Soller & White, 2003) (see also the subsequent section). A key initial insight was that ectopic expression of each of the three family members (Elav, Fne, and Rbp9) was sufficient to extend 3′ UTRs not only on individual loci (Hilgers et al, 2012; Zhang et al, 2019) but also indeed on the genome-wide scale in a non-neuronal line of cultured cells (Wei et al, 2020) (Fig 6A). These data demonstrate ELAV/Hu RBPs have intrinsic activities to rewire the 3′ landscape, independent of other features of neurons. This raised the question of whether they were endogenously responsible for this phenomenon. Indeed, although mutation of *elav* causes complete lethality at an early larval stage, their CNS is still largely able to adopt neuronal 3′ UTR extensions, whereas mutants of the other family members are relatively normal. Of note, Elav is the earliest expressed of the three ELAV/Hu RBPs, suggesting that its temporal primacy may contribute to its phenotypic requirement. By contrast, double mutants of *elav* and *fne* substantially abrogated the accumulation of neural 3′ UTR extensions (Carrasco et al, 2020; Wei et al, 2020). Thus, these factors are both necessary and sufficient to direct substantial aspects of the neural 3′ landscape. Nevertheless, when profiling dissected early larval CNS, *elav+fne* mutants still retain a subset of neural extensions. It remains to be seen whether these depend on the third member of the family (Rbp9) or perhaps other factors yet to be identified.

How do ELAV/Hu RBPs mediate this process? Based on synthetic tethering reporters, it was earlier proposed that Elav may locally inhibit proximal isoform processing by binding upstream of cleavage sites (Hilgers et al, 2012). Subsequent studies supported a potential co-transcriptional activity because ELAV/Hu-mediated 3′ UTR extension was detected in nascent, chromatin-associated transcripts. However, genomic analysis of the properties of bypassed cleavage sites indicated the presence of characteristic U-rich ELAV-type binding sites just downstream of proximal cleavage sites, concomitant with enriched binding of Elav at this location from CLIP-seq studies (Fig 6A) (Carrasco et al, 2020; Wei et al, 2020). However, as ELAV-type sites are correlated with this, but not definitively on a gene-by-gene basis, it is possible that other gene features might impact this process (Oktaba et al, 2015). Moreover, the precise molecular strategy by which ELAV/Hu RBPs inhibit CPA remains to be determined. Mammalian orthologs can interact with core CPA factors Cstf64 and CPSF160 (Zhu et al, 2007), suggesting direct inhibition; however, *Drosophila* Elav does not seem to displace CstF or CPSF (Soller & White, 2003). This topic deserves further mechanistic studies.

Notably, it still remains to be determined what factors underlie neural APA in mammals, although its members of this family (ubiquitous HuR and neural-restricted HuB/C/D) are certainly prime suspects. In fact, this family was previously shown to promote 3′ UTR extension of the HuR gene itself (Dai et al, 2012; Mansfield & Keene, 2012), and HuC was also shown to influence APA during neural differentiation (Grassi et al, 2018). Based on research with their *Drosophila* counterparts, it seems likely that mammalian ELAV/Hu RBPs may have overlapping activities. Indeed, they bind overlapping target sites (Ray et al, 2013), and available antibodies do not distinguish among HuB/C/D because of their similarity. Accordingly, analysis of the complete knockout of the ELAV/Hu family during neural specification and differentiation will certainly be instructive.

### Control of neural-specific distal ALE splicing by ELAV/Hu RBPs

As mentioned, ALE splicing regards a special subset of alternative exons, which comprise non-overlapping 3′ exon choices. Although these can be regulated at the level of splicing into different ALE sites (Yap et al, 2016), it appears that many neural ALE events are actually regulated at the level of 3′ CPA. In fact, the initial studies of Elav-mediated mRNA processing in *Drosophila* involved switching from proximal to distal ALE isoforms in neurons at the *erect wing* and *neuroglian* loci (Koushika et al, 1996, 2000; Lisbin et al, 2001). This was later found to be mediated by suppression of proximal 3′ cleavage by Elav (Soller & White, 2003).

With newer genomic profiling data, it became clear that ELAV/Hu RBPs directionally determine ALE isoforms in neurons, in a similar manner as terminal neural 3′ UTRs (Fig 6B). That is, dozens of loci switch from proximal to distal ALE isoforms in nervous system (Lee et al, 2022), and these globally retreat to proximal ALE isoforms upon combined mutation of elav/fne (Lee et al, 2021). Again, this is not simply due to global disruption of neural gene expression in these mutants because ectopic expression of each of the three ELAV/Hu RBPs was able to force “naive” *Drosophila* S2 cells into the neural distal 3′ ALE landscape (Lee et al, 2021). Alongside the other effects described for cassette exon splicing and APA, these observations establish that *Drosophila* ELAV/Hu RBPs are responsible for multiple aspects of the neural-specific alternative transcriptome.

Interestingly, recent large-scale studies of mammalian APA across numerous unrelated datasets concluded that ALE isoform bias and 3′ UTR length bias are co-regulated across the transcriptome (Goering et al, 2021), even though HuB/C/D are strongly restricted to neurons (Agarwal et al, 2021; Lee et al, 2022). Therefore, outside of the ELAV/Hu family, there may be other strategies that globally coordinate these two regimes of alternate mRNA isoform processing. Another set of candidates to mediate this joint process are MBNL proteins (Batra et al, 2014; Taliaferro et al, 2016). However, the broad co-regulation of ALE/APA programs also suggests there might be intrinsic features to the layout of metazoan genes that governs their 3′ cleavage from upstream to progressively more downstream locations along the gene model.

### Back-splicing/circular RNAs (circRNAs)

circRNAs comprise another class of unusual splice isoform, that is, especially abundant in neurons. These are typically generated by back-splicing of exons to yield circular species at the expense of linear isoforms. As circRNAs have been extensively reviewed elsewhere (Kristensen et al, 2019; Yang et al, 2022), we will only mention them briefly here. As with isoforms of protein-coding transcripts, it has become clear in the past decade that circRNAs are exceptionally diverse (Nielsen et al, 2022) and substantially expand our conceptions of alternative splicing processes (Hansen et al, 2013; Memczak et al, 2013). Early on, they were recognized to be generally much higher expressed in nervous system/neurons compared with most other cell types and tissues (Westholm et al, 2014; Rybak-Wolf et al, 2015; You et al, 2015). This may be due in part to regulation of circRNA biogenesis by alternative splicing factors, many of which are involved in neural alternative splicing (e.g., Quaking, Nova, Mbnl, FUS, and others) (Ashwal-Fluss et al, 2014; Conn et al, 2015; Errichelli et al, 2017; Knupp et al, 2021). Nevertheless, it may also be the case that the high accumulation of circRNAs in neurons may simply be due to the unusual longevity of these cells, coupled with resistance of circRNAs to exonucleolytic RNA decay pathways.

**Figure 7.**
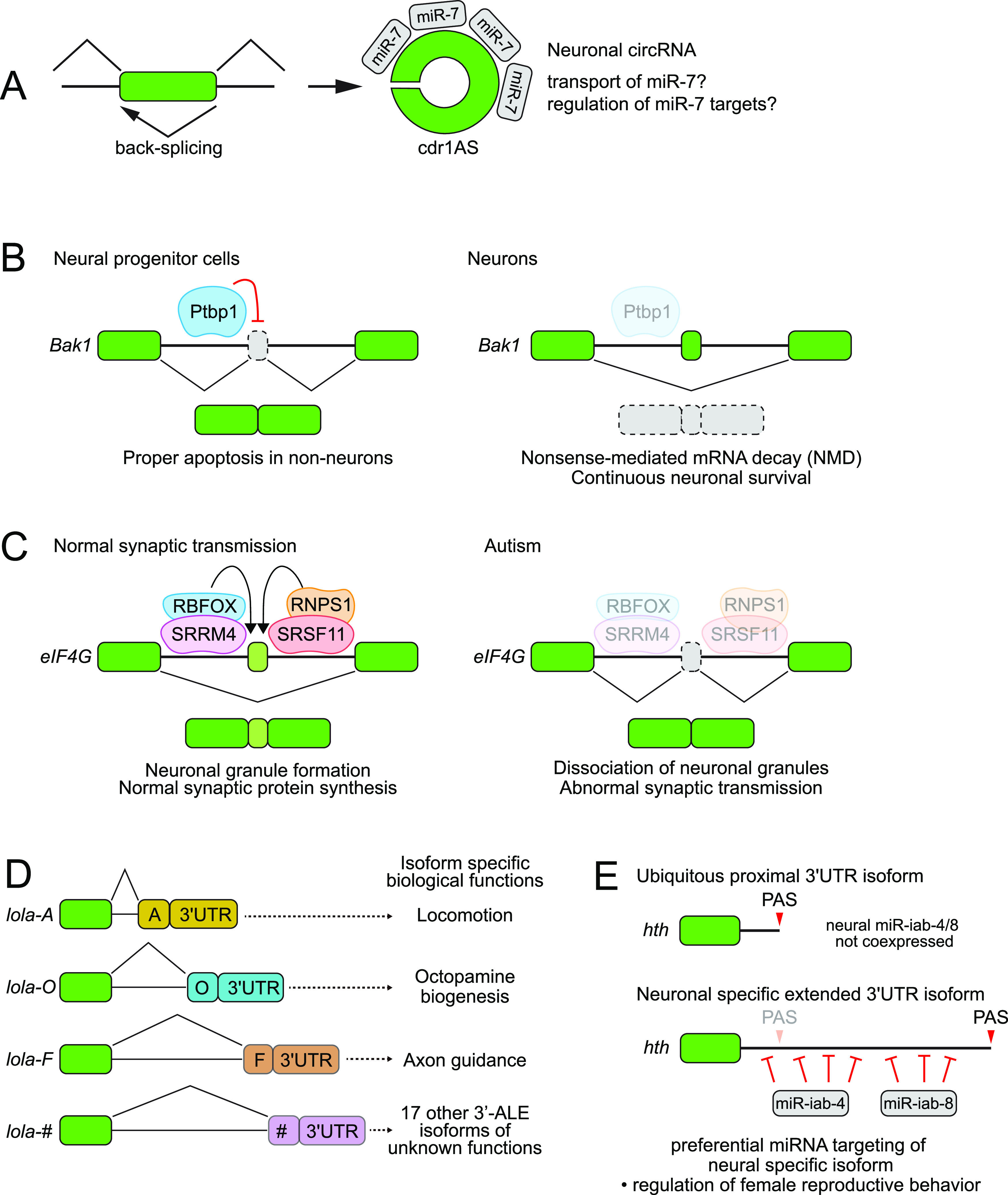
Phenotypically, significant roles for neural-specific splice and 3′ UTR isoforms. **(A)** *cdr1AS* is an abundant neural circRNA that contains >70 binding sites for miR-7. Although initially proposed as a sponge for miR-7, it. may actually facilitate miR-7 activity by transporting it within neurons. **(B)** Inhibition of neural apoptosis via microexon splicing of the core pro-apoptotic factor *Bak1*. In non-neural cells, Ptbp1 suppresses inclusion of a nonsense-encoding microexon in *Bak1*. In neurons, which lack Ptbp1, inclusion of the *Bak1* microexon leads to its degradation. Genetic deletion of the *Bak1* microexon in mice results in ectopic brain apoptosis and early lethality. **(C)** Alternative function of eIF4G via its neural-specific microexon is required for normal synaptic activity. Genetic deletion of the *eIF4G* microexon impairs neuronal function and may be a basis of autism, which exhibits preferential loss of neural microexon inclusion. **(D)** *Drosophila lola* is subject to an exceptional degree of ALE splicing, generating 20 different ALE exons, almost all of which encode distinct DNA-binding domains and all of which bear non-overlapping 3′ UTRs. Systematic deletion alleles of all *lola* ALEs identified at least three with specific, overt, phenotypic requirements. Clearly, the others must have additional as-yet-unknown functions, or there may be combinations of ALE isoforms with overlapping functions. **(E)** *Drosophila homothorax* (*hth*) encodes a transcription factor with broad roles in tissue patterning. *Hth* expresses a highly extended 3′ UTR isoform in post-mitotic neurons, which facilitates its repression in a regionally specific manner in the CNS via miR-iab-4/8 miRNAs, which are expressed in posterior segments of the ventral nerve cord. Genetic ablation of the *hth* neural 3′ UTR extension causes female virgins to adopt mated-specific behaviors, similar to specific point mutation of the miR-iab-4/8–binding sites in *hth* and deletion of the *mir-iab-4/8* locus itself.

Some circRNAs, most famously *cdr1AS* which is laden with miR-7–binding sites, have regulatory impacts in neurons (Fig 7A) (Piwecka et al, 2017; Kleaveland et al, 2018). In general, there is much to learn about the regulated biogenesis of neuronal circRNAs, and the extent to which they may have notable regulatory functions. Such insights will benefit from further knowledge as to whether circRNAs are subject to specific biogenesis pathways, which are not simply a by-product of alternative forward splicing regulation (Chen et al, 2022; Pamudurti et al, 2022). In addition, strategies to disrupt circRNA accumulation without affecting linear counterparts enable their functional study (Pamudurti et al, 2020; Li et al, 2021) and should help inform the extent to which circRNAs might harbor transacting activities (Suenkel et al, 2020; Knupp et al, 2022). Alternatively, it might be that some or many circRNAs are aberrant products (Xu & Zhang, 2021) but are tolerated as part of the inevitable noise in mRNA processing. Another possibility is that high levels of circRNAs in neurons may have deleterious effects, which may conceivably be of consequence during aging or potential neurodegeneration (Westholm et al, 2014; Gruner et al, 2016; Knupp et al, 2022).

### Neural defects caused by misregulation of individual splicing/APA events

It is clear that dysfunction of global regulators, such as RBPs that direct large-scale programs of neural-specific splicing and/or polyadenylation, can have profound phenotypic consequences (Calarco et al, 2009; Ince-Dunn et al, 2012; Licatalosi et al, 2012; Irimia et al, 2014; Zhang et al, 2016; Wei et al, 2020). However, this does not necessarily mean that the collective deregulation of all these targets is responsible for the net phenotype. Alternatively, it is plausible that mis-splicing or aberrant 3′ UTR utilization of many individual neural targets might be tolerated, whereas only the subset of isoform targets is critical. The specific delineation of phenotypically substantial isoform diversity at individual genes is challenging to obtain, but on the other hand, these have special impact for thinking about how isoform regulation may underlie disease.

The most direct evidence for the causality of isoform switching for in vivo phenotype is the generation of endogenous alleles that specifically ablate individual isoforms, or perhaps reciprocally make them constitutive. For example, *Drosophila Dscam1* can generate ∼38,000 isoforms of cell adhesion molecules that play diverse roles in neuronal wiring (Schmucker et al, 2000). Indeed, complex genetic engineering demonstrates that thousands of *Dscam1* isoforms are minimally required for normal neural patterning (Chen et al, 2006; Hattori et al, 2007). However, it is useful to bear in mind that such genetic manipulations are time-consuming and come with some practical risks for individual researchers: what if there is only negative data result from years of investigation? The “survivorship bias” of this type of study is worth noting because despite increasing opportunities to report negative results, they are generally under-recognized.

It might be that phenotypes of some “executive” regulators, such as splicing and APA factors, cannot be recapitulated until a large cohort of targets is simultaneously mutated. However, as it is currently not feasible to conduct such experiments in vivo, most reports of the “network” scenario are strictly correlative. On the other hand, there are a growing number of genetic studies that definitively show that loss of individually regulated splicing and/or 3′ UTR events cause mutant phenotypes. We highlight a few recent examples that exploit precision genetics in intact animals to delineate phenotypic requirements for individual alternative mRNA-processing events in neurons.

#### Inhibition of neural apoptosis via alternative poison–exon splicing of Bak1

One of the many distinguishing features of neurons is their longevity. Because capacity for adult neurogenesis is spatially restricted and/or limited in many animals, it is important to preserve neurons throughout adult life (Obernier & Alvarez-Buylla, 2019). In fact, the survival of mammalian neurons seems to be limited by their host, and they can live much longer in transplants (Magrassi et al, 2013). Are there specific regulatory mechanisms that prevent neuronal death? Careful genetic studies link neuronal-specific splicing regulation of the central pro-apoptotic factor Bak1 with suppression of neuronal cell death.

Following appropriate genetic and/or environmental stimuli, the BH domain factors Bak1 and Bax form hetero- or homo-oligomers that form mitochondrial pores, leading to activation of executioner caspases. Not only is *bak1* alternatively spliced, it is processed to include a microexon specifically in neurons. Interestingly, the 20 nt microexon is highly conserved across mammals but contains an invariant stop codon, rendering it an NMD substrate. The inclusion of this alternative *bak1* exon is repressed by PTBP1 (Lin et al, 2020), which is broadly expressed but lost in neurons (Keppetipola et al, 2012). Accordingly, although neural progenitor cells express Bak1 protein, as with non-neural cell types, neuronal *bak1* transcripts preferentially include the microexon and do not yield Bak1 proteins (Fig 7B) (Lin et al, 2020).

To address whether *bak1* alternative splicing has in vivo consequences, the neural microexon was deleted to yield *Bak(∆MIC)*. Astonishingly, this allele is homozygous lethal at early postnatal stages, accompanied by selective misexpression of Bak1 proteins in the brain and ectopic neural apoptosis (Lin et al, 2020). Thus, among thousands of neural alternative splicing events, the appropriate processing of this individual neural splice isoform is truly a life or death situation.

#### Neuronal splicing of eIF4G factors is required for normal synaptic activity and social behavior

Building on the finding that neuronal microexons are generally misregulated in autism (Quesnel-Vallieres et al, 2016; Gonatopoulos-Pournatzis et al, 2018), it becomes pressing to know if individual microexons are required for neural development or function. A recent study now followed through with rigorous functional and genetic analysis. Among loci with neural-specific microexons are translation initiation factors eIF4G1 and eIF4G3. Knockout mice were engineered for both microexons, yielding viable, overall normal animals (Gonatopoulos-Pournatzis et al, 2020a). However, *eIF4G1(∆MIC)* animals exhibit selective behavioral alterations in social paradigms, along with increased transmission at inhibitory synapses and increased levels of certain synaptic proteins (Fig 7C). These phenotypes support the notion that disruption of human eIF4G microexons may predispose to autistic syndrome. Mechanistically, how could eIF4G microexons affect the function of these essential, ubiquitous factors? Interestingly, their microexons overlap prion-like domains in these factors, and their inclusion enhances phase separation of eIF4G1 in vitro and association with cytoplasmic mRNP granules in cells. Biochemical and profiling evidence also presented that the eIF4G1(MIC) isoform may enhance ribosome stalling in concert with FMRP, consistent with the increase in certain synaptic proteins in *eIF4G1(∆MIC)* mutants (Gonatopoulos-Pournatzis et al, 2020a).

Together, this work provides a converse situation to the *Bak(∆MIC)* example. Although alternative splicing of the former effectively silences this isoform in neurons via NMD, alternative splicing of the latter alters the functionality of neural eIF4G isoforms, outlining a paradigm by which the function of essential, ubiquitous factors can be modulated in a cell-specific manner.

#### Distinct functions for multiple neural ALE isoforms of lola

The *Drosophila longitudinals lacking* (*lola*) locus is required in several tissue settings, but *lola* mutants were first isolated on the basis of neural differentiation defects (Giniger et al, 1994) and it has since been found to have diverse neural functions. *lola* turned out to be subject to a high degree of alternative processing, including multiple alternative 5′ exons and 3′ exons, generating ∼80 different transcripts. A special feature of these isoforms is the high degree of functional diversity generated by ALE isoforms: of 20 different last exons, 17 encode C-terminal zinc fingers (Goeke et al, 2003; Ohsako et al, 2003). Thus, this single locus generates a multitude of transcription factors.

Most studies of *lola* used mutants that disrupt the entire locus. In a heroic effort, a high-resolution genetic dissection of the in vivo requirements of individual *lola* ALE isoforms was conducted by deleting each *lola* ALE isoform (Fig 7D) (Dinges et al, 2017). Some lola isoforms lacked apparent defects, but five were developmentally lethal and three others showed adult cytological and/or behavioral phenotypes. Detailed analyses showed how individual lola ALE isoforms mediate diverse biology. For example, lola-F mediates axonal guidance, and mutants of this single isoform recapitulate defects originally observed for the entire locus. On the other hand, lola-O prevents neural degeneration via regulation of octopamine biogenesis, whereas lola-A and lola-H are involved in locomotion (Fig 7D).

With these systematic separations of function alleles for the array of lola isoforms, many questions remain. How is the alternative processing of the distinct lola ALE isoforms regulated, and are they co-expressed or exhibit cell-specific deployment? Because regulated ALE isoforms studied to date involve binary choices, it will be fascinating to know how the appropriate *lola* ALE isoforms can be selected. Moreover, because all 20 *lola* ALE isoforms are paired with completely different 3′ UTRs, is there similar complex post-transcriptional regulation for these ALE isoforms? Finally, relevant to lola biology, are there both overlapping and/or distinct transcriptional targets for the 19 ALE Lola proteins? All of these remain to be elucidated.

#### Tandem 3′ UTR isoforms confer distinct neural regulation and behavioral function for homothorax

Many hundreds of genes express substantially elongated 3′ UTRs specifically in neurons, implying that numerous genes are subject to specialized post-transcriptional regulation in this cell type. Although these neural 3′ UTR collectively bear numerous conserved miRNA and RBP sites (Miura et al, 2013; Sanfilippo et al, 2017), until such 3′ UTR extensions have been mutated, we cannot know how important neural APA is for normal phenotype. To date, only a few genetic studies have demonstrated in vivo consequences upon targeted disruption of a neural 3′ UTR extension. For example, the long 3′ UTR of *Drosophila Dscam1* is required for specific aspects of neural differentiation and is furthermore linked to alternative cassette exon splicing of the same gene (Zhang et al, 2019). As well, the neural 3′ UTR extension of *CaMKII* is needed for local translation to control spontaneous vesicle release at the neuromuscular junction (Kuklin et al, 2017), whereas the neural 3′ UTR of *prospero* is required for mRNA accumulation via the SYNCRIP RBP (Samuels et al, 2020). As an example from mice, deletion of the neural 3′ UTR extension of *Calmodulin 1* (*Calm1*) impairs certain aspects of neural development and function (Bae et al, 2020).

Because of their high-sequence information, miRNA-binding sites are the most extensive class of functional regulatory motif to differ between APA isoforms (Sandberg et al, 2008). Accordingly, substantial numbers of conserved miRNA sites are gained in 3′ UTR extensions of neural isoforms, which could in principle selectively repress these isoforms in neurons (Smibert et al, 2012; Miura et al, 2013; Sanfilippo et al, 2017; Lee et al, 2022). Nevertheless, as lengthened 3′ UTR isoforms do not seem to be strongly biased for lower accumulation (Lianoglou et al, 2013; Wei et al, 2020), there is not a clear expectation that a gain of miRNA targeting is the predominant or only outcome for neural 3′ UTR lengthening. For example, there could also be positive regulatory elements revealed in these longer isoforms. Nevertheless, a particularly notable example of in vivo APA biology is in fact mediated by a gain of neural-specific miRNA sites by a neural isoform.

The *Drosophila* bithorax complex (BX-C) houses one of the Hox gene clusters, and notably contains three homeobox genes that govern abdominal segment identities (Lewis, 1978). It also contains *mir-iab-4/8*, which is the archetype of a bidirectionally transcribed and processed miRNA locus (Garaulet & Lai, 2015). Both miR-iab-4 and miR-iab-8 miRNAs are capable of inducing homeotic transformations of external segments when misexpressed (Ronshaugen et al, 2005; Stark et al, 2008; Tyler et al, 2008), but a specific deletion of the miRNA hairpin region (hereafter termed *∆mir*) exhibits normal external patterning (Bender, 2008). However, *∆mir* animals are far from normal because they are completely sterile in both sexes and exhibit defective reproductive behaviors (Bender, 2008). In particular, *∆mir* virgin females exhibit a subjective behavioral shift, and instead perform a suite of behaviors characteristic of the mated state (Garaulet et al, 2014). Although *∆mir* derepresses direct BX-C targets Ubx and Abd-A in abdominal regions of the ventral nerve cord (equivalent of the vertebrate spinal cord), the most overtly misexpressed protein is the Hox cofactor homothorax (Hth) (Garaulet et al, 2014, 2020).

Interestingly, not only is *hth* is predicted to be heavily targeted by conserved binding sites for both miR-iab-4/8, many of these are located in its neural-specific 3′ UTR extension (Fig 7E). To determine if these directly mediate Hth repression *in cis*, CRISPR was used to generate an engineerable allele of the *hth* 3′ region, which could be replaced with mutagenized derivatives. miR-iab-4/8 sites were directly required to suppress Hth protein expression in the ventral nerve cord, as *hth(∆miR)* mutant virgin females also adopt mated behaviors. Moreover, this could be directly traced to failure of neural APA-mediated repression because deletion of the *hth* neural 3′ UTR (but not its universal 3′ UTR) largely phenocopied the *hth(∆miR)* allele (Garaulet et al, 2020). Altogether, precise genomic engineering demonstrates how neural APA enables miRNA-mediated spatial restriction of a homeotic transcription factor, whose misexpression alters internal behavioral state and reproduction.

### Future prospects

The neural transcriptome is highly diversified in multiple ways that are unique to this celltype and likely underlie the unique attributes and properties of neurons. Although the community has made great insights into how this is accomplished, we are still some ways from a complete understanding of its complexity. Even though there has been great progress using deep learning to predict the “splicing code” (Baeza-Centurion et al, 2019; Jaganathan et al, 2019), we are evidently not as knowledgeable as cells themselves, which must make the appropriate processing choices without aid of computer servers. Deeper annotation of rare and/or neural subtype-specific isoforms may come with the burgeoning amount of single-cell data, including whole transcriptome datasets (Feng et al, 2021; Olivieri et al, 2021). In parallel, it is worthwhile to have more comprehensive functional discovery of isoform regulators. Beyond general CRISPR-library and ORF-library screening strategies, reagents exist now for systematic tethering of all RBPs (Luo et al, 2020). Applied to the right reporter systems, one can easily imagine that additional isoform regulators can be deduced. A more thorough annotation of isoforms and their regulations may permit improved predictions of the regulatory consequences of cis-encoded variants and mutations (Chiang et al, 2022), perhaps in concert with expression changes in trans-encoded regulators (Agarwal et al, 2021; Lee et al, 2022).

Most of the mechanisms discussed in this review regard strategies by which the selection of alternative splicing sites, and of APA sites, is executed locally and independently. If and how these isoform programs are coordinated is not fully understood, but the application of long-read sequencing permits coordinated splicing events (Joglekar et al, 2021; Glinos et al, 2022) and coordination of splicing and 3′ end formation (Zhang et al, 2019; Reimer et al, 2021) to be identified. Moreover, recent studies reveal mechanistic linkages between alternative splicing and 3′ end formation (Zhang et al, 2019; Lee et al, 2021), splicing and promoter choice (Tasic et al, 2002; Fiszbein et al, 2019), alternative promoter choice and alternative 3′ end formation (Alfonso-Gonzalez et al, 2023), and transcriptional regulation and/or elongation rate with alternative splicing and/or 3′ end formation (Muniz et al, 2021; Geisberg et al, 2022; Kwon et al, 2022). Recent technical innovations should enable more comprehensive understanding of the transcriptome. In particular, methods that combine single-cell sequencing and long-read strategies can resolve connectivity of individual alternative exons of individual cells (Singh et al, 2019; Fan et al, 2020; Philpott et al, 2021; Hardwick et al, 2022).

Finally, it is worth considering whether aspects of documented isoform complexity might reflect errors (Saudemont et al, 2017; Xu & Zhang, 2021). Although these might still be cataloged using deep datasets, they may or may not have any physiological consequence, especially if such isoforms are rare. But what about the more abundant neural-specific isoforms that involve alternative splicing or 3′ UTRs? If they are exquisitely regulated, we may presume their biological significance. Yet there is a strong difference between molecular validation and phenotypic impacts. Ultimately, we seek to define the subset of neuron-specific isoforms that are most relevant to disease. The examples discussed involved laborious genetic engineering and are poorly suited to comprehensive studies. Although there can be no substitute for careful genetics, it is nonetheless enticing to consider if there are more facile strategies to obtain candidate hits. Recently, the first successful CRISPR-based exon deletion screens were reported (Gonatopoulos-Pournatzis et al, 2020b; Thomas et al, 2020). Although these were initially applied to immortalized cancer cell lines, it is straightforward to imagine that they could be in vitro–differentiated neurons, to screen for functional consequences of neural-specific alternative internal exons, last exons, and/or 3′ UTRs. We can only dream of the results of such tests, but the tools are now ready and waiting for us.
